# RHPCG: a database of the Regulation of the Hippo Pathway in Cancer Genome

**DOI:** 10.1093/database/baz135

**Published:** 2019-12-17

**Authors:** Chengyu Wang, Fan Yang, Tingting Chen, Qi Dong, Zhangxiang Zhao, Yaoyao Liu, Bo Chen, Haihai Liang, Huike Yang, Yunyan Gu

**Affiliations:** 1 College of Bioinformatics Science and Technology, Harbin Medical University, Baojian Road, Nangang District, Harbin, 150086, China; 2 Department of Pharmacology, Harbin Medical University, Baojian Road, Nangang District, Harbin 150086, China; 3 Department of Anatomy, Harbin Medical University, Baojian Road, Nangang District, Harbin 150086, China

**Keywords:** Hippo signaling pathway, Cancer genome, Regulatory motif, The Cancer Genome Atlas

## Abstract

The Hippo signaling pathway is a highly conserved pathway controlling organ size, cell proliferation, apoptosis and other biological functions. Recent studies have shown that Hippo signaling pathway also plays important roles in cancer initiation and progression. However, a database offering multi-omics analyses and visualization of Hippo pathway genes in cancer, as well as comprehensive Hippo regulatory relationships is still lacking. To fill this gap, we constructed the Regulation of the Hippo Pathway in Cancer Genome (RHPCG) database. Currently, RHPCG focuses on analyzing the 21 core Hippo-protein-encoding genes in over 10 000 patients across 33 TCGA (The Cancer Genome Atlas) cancer types at the levels of genomic, epigenomic and transcriptomic landscape. Concurrently, RHPCG provides in its motif section 11 regulatory motif types associated with 21 core Hippo pathway genes containing 180 miRNAs, 6182 lncRNAs, 728 circRNAs and 335 protein coding genes. Thus, RHPCG is a powerful tool that could help researchers understand gene alterations and regulatory mechanisms in the Hippo signaling pathway in cancer.

## Introduction

The Hippo signaling pathway is a highly conserved pathway initially discovered in Drosophila (1) and consists of a group of kinases, including among others the serine/threonine STE20-like protein kinases (serine/threonine–protein kinase 3 (STK3), also known as MST2, and serine/threonine-protein kinase 4 (STK4), also known as MST1), the large tumor suppressors (large tumor suppressor kinase 1 (LATS1) and 2 (LATS2)), and the MOB kinase activators (MOB1A/MOB1B) (2). These kinases directly or indirectly phosphorylate the key function effectors, yes-associated protein (YAP) and transcriptional coactivator with PDZ-binding motif (TAZ), thus blocking the transcription of downstream target genes.

As we known, the Hippo signaling pathway has a major role in the regulation of cell proliferation, apoptosis, migration and differentiation, involved in organ development (3). Dysregulation of the Hippo signaling pathway, or alterations of pathway genes, has been shown to result in several diseases, including fibrosis, cardiopathy and cancer (4–6). Many recent studies have revealed that Hippo signaling pathway plays key roles in cancer initiation and progression, such as liver, colorectal, gastric, prostate and other types of cancer (7–10). The oncogenic or tumor suppressor function of Hippo-signaling-pathway genes has also been reported. Hippo-signaling-pathway genes have been demonstrated to exhibit alterations in cancer samples at the levels of genome, epigenome, transcriptome and proteome (11). The *YAP1* and its paralog *TAZ* have been reported to function as oncogenes in various malignant tumors. Specifically, *YAP1/TAZ* have been shown to play the role of transcriptional coactivators with the TEA-domain DNA-binding family of transcription factors (TEADs) during carcinogenesis, including cell proliferation, epithelial-mesenchymal transition (EMT) and apoptosis. Overexpression and activation of *YAP1* has been involved in the development of gastric cancer (12, 13). The salvador family WW domain containing protein 1 (*SAV1*) is the human homologue of the Salvador protein and was reported to repress human colorectal cancer by inhibiting the Akt-mTOR signaling pathway (14, 15). Accordingly, downregulation of *SAV1* promoted the growth process of colon cells (14, 15). However, and despite the accumulated knowledge, a database systematically analyzing and presenting the deregulation of Hippo-signaling-pathway genes and its effects in pan-cancer at the levels of genome, epigenome, transcriptome and proteome is still lacking.

Meanwhile, Hippo signaling pathway is known to co-operate with other signaling pathways in carcinogenesis (16). Hippo signaling pathway has been shown to regulate other pathway switches mediated by regulatory factors, such as transcription factors (TF), microRNAs (miRNA), long non-coding RNAs (lncRNA) and circular RNAs (circRNA) at the transcriptional or post-transcriptional levels (17, 18). For example, the SNHG15 lncRNA has been reported to act as a tumor promotor in many types of cancer. A recent study demonstrated that SNHG15 served as a competitively endogenous RNA (ceRNA) in regulating *YAP1* in Hippo signaling pathway by sponging miR-200a-3p in papillary thyroid carcinoma (19). These regulators interact with Hippo genes through the basic pattern of motifs in the regulatory network, such as the ceRNA pattern (20).

Here, we developed a database, named **R**egulation of the **H**ippo **P**athway in **C**ancer **G**enome (RHPCG) as an open and up-to-date online manually curated depository of molecular alterations and interactions of the Hippo-signaling-pathway genes. The RHPCG database collected six types of regulatory ships among protein coding genes, microRNAs, lncRNAs and circRNAs from 12 public databases and integrated them into 11 diverse motifs. In addition, RHPCG systematically analyzed the multi-omics data obtained from The Cancer Genome Atlas (TCGA) containing genomic, proteomic, epigenomic and transcriptome data. Meanwhile, the RHPCG database provides a user-friendly web interface that allows users to browse, search and download the gene alterations of Hippo signaling pathway in pan-cancer at multiple levels of omics, as well as the regulatory motifs associated with Hippo signaling pathway.

## Materials and Methods

### Molecular data in RHPCG

Protein-coding genes of *Homo sapiens* were collected from the NCBI database (21). Likewise, lncRNAs, including ‘lincRNA’, ‘antisense’, ‘processed transcript’, ‘sense intronic’ and ‘To be Experimentally Confirmed (TEC)’ were obtained from Gencode (Release 24) (22). Mature and precursor miRNAs with ID were downloaded from miRBase (Release 21) (23). The genomic position and the best transcription of circRNAs were collected from circBase (24). All gene symbols used in RHPCG conformed to The **HUGO Gene Nomenclature Committee** (HGNC) guidelines and Hippo-signaling-pathway genes were obtained from Kyoto Encyclopedia of Genes and Genomes (KEGG).

### Multi-omics data of pan-cancer

**Figure 1:**
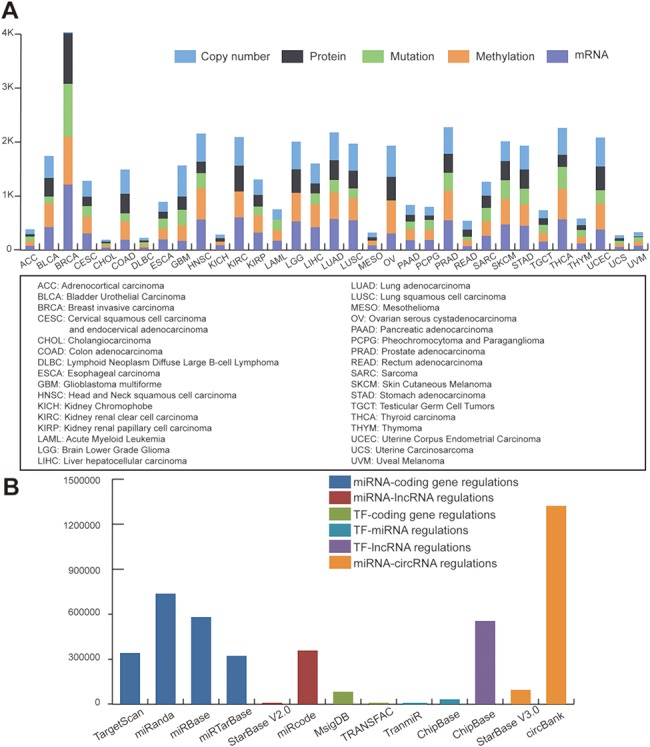
Statistics of data sources. (A) Statistics of TCGA multi-omic datasets across pan-cancer. (**B**) Statistics of regulatory patterns derived from databases.

We downloaded TCGA multi-omics datasets from GDAC firehose (http://gdac.broadinstitute.org/), containing over 10 000 samples and 33 cancer types (Figure 1A). The log2 transformed expression levels were used to preprocess the TCGA mRNA expression profiles. Regarding the mutation dataset, we selected the non-silent mutation type. Level 3 of the DNA copy number datasets of the Genome-Wide Human SNP Array 6.0 platform were obtained from TCGA. Using the cBioPortal definitions (25), copy number alterations were classified as gene amplifications, gains (low-level amplifications), deep deletions (equivalent to homozygous deletions for non-aneuploidy cases) and shallow deletions (heterozygous loss). For methylation data, we used the beta value of methylation to represent the levels of DNA epigenetic changes.

### Potential targets of Hippo-signaling-pathway core genes

We downloaded the gene-interaction relationship dataset from Pathway Commons (http://www.pathwaycommons.org/). Genes with interactions with the Hippo-signaling-pathway-core genes constituted the potential targets. For the target gene differential expression test, we used the *t*-test to evaluate whether potential targets were differentially expressed between samples of the high-expression group and low-expression group divided by the mid-value of mRNA expression of Hippo core gene based on TCGA cancer expression profiles.

### Regulatory data for motif definition

To define regulatory motifs, we comprehensively collected six regulatory patterns from 12 public databases: miRNA-coding gene regulatory patterns obtained from TargetScan (26), miRanda (27), miRbase (28) and miRTarBase (29); miRNA-lncRNA interactions obtained from StarBase V2.0 (30) and miRcode (31); TF-gene interactions collected from MsigDB (32) and TRANSFAC (33); TF-miRNA data downloaded from TransmiR (34) and ChipBase (35); TF-lncRNA interactions obtained from ChipBase; and miRNA-circRNA interactions collected from circbank and StarBase V3.0. Statistics of the regulatory data are shown in Figure 1B.

### Data integration

The interactions of TF-target, miRNA-target, miRNA-lncRNA, TF-lncRNA, TF-miRNA and miRNA-circRNA, collected from public databases (Supplementary Table 1), were integrated into a regulatory network. Then, we searched for 11 types of motifs in the integrated regulatory network, including both the loop and linear motifs. In our work, we only focused on the basic motifs, which consist of three nodes (Table 1).

**Table 1:** Details of the 11 types of motifs in RHPCG

Type	Motifs	Details
1	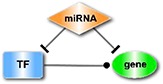	The miRNA-FFL (feed-forward loops), in which a miRNA is the master regulator.
2	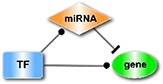	The TF-FFL, where a TF regulates the partner miRNA and their common target.
3	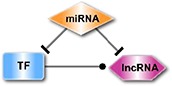	The miRNA-mediated loop, in which an lncRNA is the joint target of both the miRNA and TF.
4	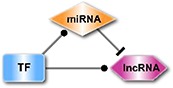	The TF-mediated loop, in which an lncRNA is the common target of both the TF and miRNA.
5	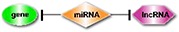	The line in which ceRNAs or lncRNAs can regulate the expression of protein-coding genes by competing for common miRNA response elements at post-transcriptional level.
6	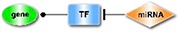	The TF-mediated line in which a protein-coding gene is the target.
7	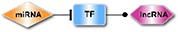	The TF-mediated line in which an lncRNA is the target.
8	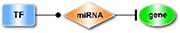	Feedback loops (FBLs) in which a TF activates transcription of a miRNA, which in turn inhibits the translation of the TF.
9	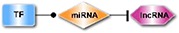	The miRNA-mediated line in which an lncRNA is the target.
10	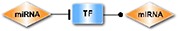	Feedback loops in which a miRNA inhibits translation of a TF, which in turn activates the transcription of the TF.
11	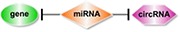	The miRNA-mediated ceRNA in which a circRNA is the competing endogenous RNA for the target gene.

### Construction of RHPCG

The Linux, Apache, MySQL, and Python (LAMP) architecture were implemented to build the dynamic database backend. The RHPCG database was built on an Apache HTTP server (version 2.4.6) installed on a machine with CentOS (version 7.2.1511) as the operating system. All data in RHPCG had been organized using MySQL (5.6.34). The website was developed based on Django and HTML. Web components were designed in Cascading Styling Sheet (CSS) and Javascript (JS), and tested in the latest versions of Google Chrome (version 76.0.3809.100) and Firefox (version 68.0.1) browsers.

## Results

### Mechanism of Hippo signaling pathway in cancer

**Figure 2:**
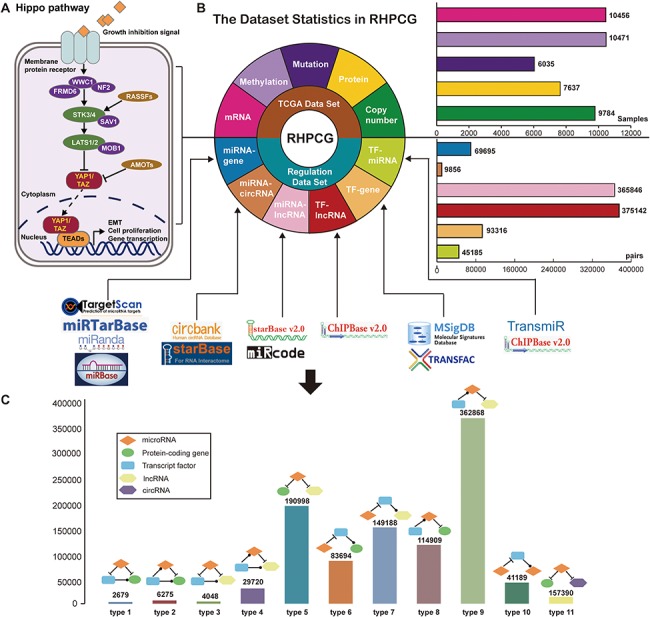
Flow chart of the RHPCG database. (**A**) Core genes in the Hippo signaling pathway. (**B**) Dataset statistics in RHPCG. (**C**) Statistics of regulatory motifs in RHPCG.

In general, we divided the Hippo signaling pathway into three main parts: (1) upstream regulators (neurofibromin 2 (*NF2*), WW and C2 domain containing 1 (*WWC1*), FERM domain containing 6 (*FRMD6*)), (2) core kinase molecules (*STK3/4, LATS1/2, SAV1, MOB1*) and (3) major co-activators (*YAP1/TAZ*) (Figure 2A). The membrane receptor transmits extracellular inhibitory signals to upstream factors leading to a series of kinase phosphorylation reactions. The outcome of the Hippo signaling pathway has been shown to be the cytoplasmic retention and eventual degradation of the *YAP1* and *TAZ* coactivators (36). In contrast, when the Hippo signaling pathway is inhibited, the *YAP1/TAZ* coactivators go into the nucleus and bind to TEADs to start the downstream transcription process, which induces EMT and cell proliferation (Figure 2A). Based on previous literature supporting evidence, RHPCG manually defined 21 ‘core genes’, which play essential roles in the Hippo signaling pathway (Supplementary Table 2).

### RHPCG: database content

In its current version, RHPCG has collected data from over 10 000 patients across 33 TCGA cancer types at the levels of genomic, epigenomic and transcriptomic landscape (Figure 1A). Meanwhile, RHPCG contains 180 miRNAs, 6182 lncRNAs, 728 circRNAs and 335 coding genes. Concurrently, in the motifs section, RHPCG contains six interaction types (Figure 2B) in 11 basic motifs present in signaling regulatory networks. Motifs with at least one of the 21 core Hippo genes were defined as Hippo-related motifs in the RHPCG database. The statistics of motifs are shown in Figure 2C.

### RHPCG: introduction of interfaces

The RHPCG database provides a user-friendly web interface that allows user to browse, search, and download alterations of Hippo-signaling-pathway genes in pan-cancer at multiple levels of omics, as well as the regulatory motifs associated with the Hippo signaling pathway.

**Figure 3:**
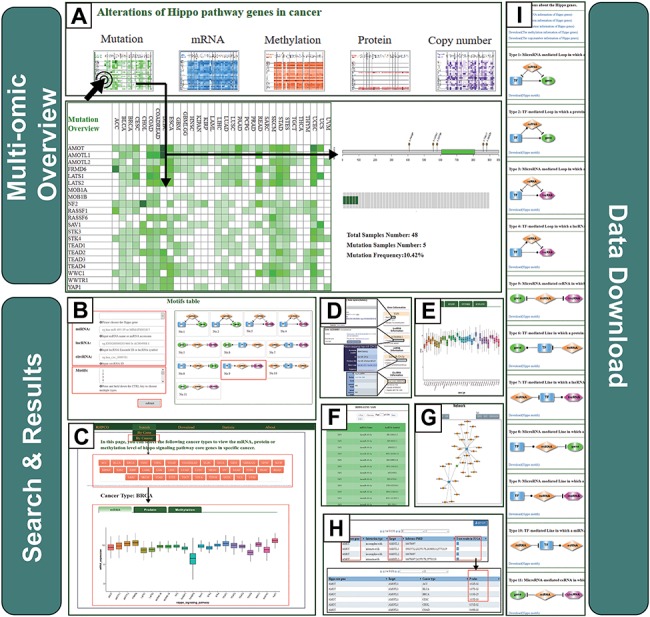
Operating instructions of the RHPCG database. (**A**) Overview of the multi-omics interface of RHPCG. (**B**) Search by gene section. (**C**) Search by cancer section. (**D**) Information box. (**E**) Multi-omics visualization section. (**F**) Table of motif results. (**G**) Network of motifs. (**H**) Potential targets of the Hippo signaling pathway. (**I**) Download page of RHPCG.

The RHPCG interface has two distinct searching sections, either searching by genes or by cancer type. On the top of the ‘searching by gene’ page, the multi-omics overview section provides an overview of the alterations of Hippo core genes across 33 cancer types at different levels of omics (Figure 3A). At the bottom of the search page, RHPCG provides a search section (Figure 3B). Users can select a gene from the list of the Hippo pathway core genes. The Ensembl ID or symbol of lncRNAs are also acceptable. Regarding miRNAs the search condition only supports search by miRNA name, whereas for circRNAs it only supports search by circRNA ID (Figure 3B). In addition, all 11 types of motif are listed on the right (Figure 3B). Users can choose one or more specific motif types for a more precise result. Additionally, we built a section in the database to enable users to search by cancer types (Figure 3C). In this section, users can select a specific cancer (Figure 3C) and view the mRNA expression, protein expression and DNA methylation of all Hippo core genes (Figure 3C).

In a similar manner, search results were divided into three main parts: The first part is the information box. The box contains the gene symbol, Entrez ID with link to source website, the location in the chromosome and a brief description of the function of the gene (Figure 3D). In the case of an lncRNA, the box shows the Ensembl ID, symbol and type (lincRNA, antisense, sense intronic, etc.) of the lncRNA (Figure 3D). For a miRNA, the box contains the accession number and mature sequence obtained from miRBase (Figure 3D). Regarding a circRNA, the box contains the best transcript, annotation and sequence from circBase (Figure 3D). The information box also contains external links to NCBI, miRbase, Ensemble or circBase for more details (Figure 3D). The second part is the visualization of the mutation frequency, copy number alteration, mRNA expression, protein expression and methylation level in 33 cancer types (Figure 3E). In the third part, all the motifs containing at least one of the gene/miRNA/lncRNA/circRNA for which the users inquired are listed in the table of motifs (Figure 3F). Accordingly, the results of each motif type could be visualized as a network. The network presentation at the bottom of the search result page can be adjusted by the legends on top of the display board. Users can also restructure the presentation themselves. The roller controls the size of the picture, and the node can be towed to the site the user desires. The RHPCG database allows users to download the search results as either a list of motifs or a network graph (Figure 3G).

Concomitantly, RHPCG enables users to view the potential targets of Hippo-signaling-pathway genes (Figure 3H). These interaction genes, which could constitute potential targets of the Hippo signaling pathway, were collected from the Pathway Commons database (http://www.pathwaycommons.org) (Figure 3H). We used the *t*-test to examine whether these potential target genes were differentially expressed between cancer samples with high and low expression of Hippo pathway genes. Consecutively, cancer samples were grouped according to the mid-value of the mRNA expression of Hippo pathway core genes (Figure 3H). Obtained *P*-values represented statistical significance (Figure 3H).

In the ‘Download’ page, users can download multi-omics datasets of Hippo-signaling-pathway genes, direct target interactions and all information on the 11 motif types. Users can conveniently access the link by clicking on the navigation panel on the right (Figure 3I).

### RHPCG: details of motifs

We integrated all interactions of TF-target, miRNA-target, miRNA-lncRNA, TF-lncRNA, TF-miRNA and miRNA-circRNA as a huge regulatory network.

**Figure 4:**
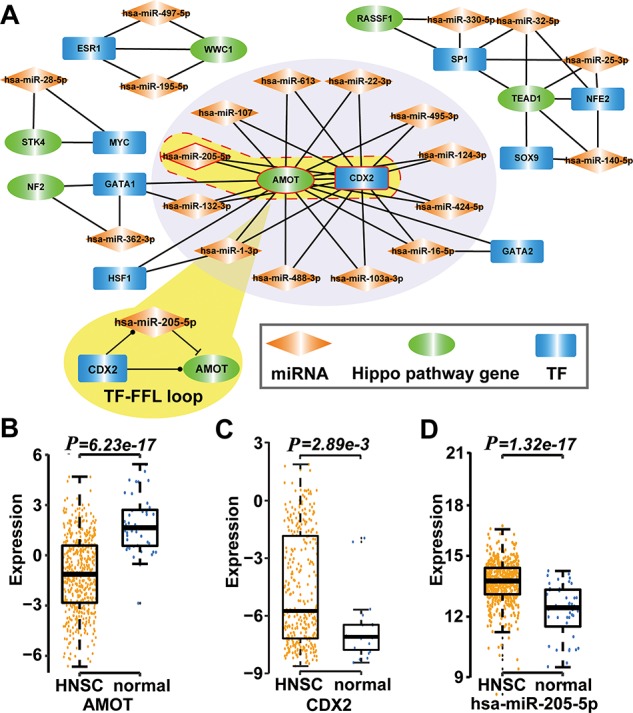
Type 2 motif (FFLs) subnetworks predicted by RHPCG. (**A**) Both *AMOT* and *CDX2* are hubs in the type 2 motif (FFLs) subnetworks. (**B**–**D**) Box plot of expression of molecules in healthy and patients with HNSC.

Then, we searched for 11 types of motifs in the integrated regulatory network. The details of these motifs are given in Table 1. Collected motifs contained at least one of the Hippo-signaling-pathway-core genes. All these data were incorporated into the RHPCG database. Consecutively, RHPCG can provide a set of convenient tools that helps researchers to easily explore the mechanisms of regulation of the Hippo signaling pathway. For example, feed-forward loops (FFLs) represent a popular type of motif in the regulatory network, within which a TF regulates a miRNA or a miRNA represses a TF, and both of them regulate a common target. By analyzing FFLs in the Hippo regulatory network, we found that angiomotin (*AMOT*) and caudal type homeobox 2 (*CDX2*) were the hubs in the FFLs formed subnetwork (Figure 4A). In particular, *AMOT* appears to be a key upstream regulating factor in the Hippo signaling pathway. In breast cancer, hsa-miR-205-5p directly inhibits *AMOT* by binding to its 3′-untranslated region (3′-UTR) (37). Accordingly, *CDX2* has been reported to represent another crucial downstream gene of the Hippo signaling pathway (38). The *CDX2* protein, a member of the caudal-related homeobox transcription factor gene family, has been proved to exert regulating roles on many miRNAs, including hsa-miR-205-5p (39). We then explored their regulatory mechanisms based on the mRNA expression profiles of TCGA cancers. In the case of Head and Neck squamous cell carcinoma (HNSC) for example, *CDX2* and hsa-miR-205-5p were significantly upregulated in HNSC compared with controls (*P* = 2.89 × 10^−3^ for *CDX2*, *P* = 1.30 × 10^−17^ for hsa-miR-205-5p; *t*-test), whereas *AMOT* was significantly downregulated in HNSC (*P* = 6.23 × 10^−17^, *t*-test) (Figure 4B-D). More specifically, the expression of hsa-miR-205-5p and that of *AMOT* showed a significantly negative correlation (*P* = 2.89 × 10^−3^, r = 0.38; Pearson correlation test). Thus, we inferred that hsa-miR-205-5p and *CDX2* coordinately regulate *AMOT* in the development of HNSC, which warrants for further biological experiments required for validation.

## Discussion

It is well known that the Hippo signaling pathway plays important roles in tumor initiation and development. Through a series of kinase cascades, the Hippo signaling pathway activates the *YAP1/TAZ* coregulators, which in turn affect downstream transcription processes, such as EMT and cell differentiation. Many studies have explored the regulatory patterns and molecular changes of the Hippo signaling pathway in cancer. Motifs, such as FFLs and ceRNAs, have been shown to be the basic patterns in the regulatory networks of tumors. To facilitate researchers in exploring the regulatory mechanisms of genes in the Hippo pathway and to further reveal the molecular alterations in multi-omic levels of the Hippo pathway genes, we integrated data from 12 public databases and analyzed TCGA multi-omics datasets to construct the RHPCG database.

Dysregulation of the Hippo signaling pathway was found in several types of cancers, suggesting that the Hippo signaling pathway might constitute an effective target for cancer treatment (11). Most studies have only focused on analyzing individual components of the Hippo signaling pathway in a single cancer type. Systematic analysis of the molecular alterations of the Hippo signaling pathway in pan-cancer at multi-omic levels is rare. Concurrently, there is a lack of knowledge regarding the regulatory relationship between the Hippo signaling pathway and other regulatory factors, including transcriptional and post-transcriptional regulation. Hence, to provide a convenient and effective way for molecular biologists and cancer researchers to investigate the Hippo signaling pathway, we constructed a database with comprehensive Hippo regulatory relationships and alterations of Hippo-signaling-pathway genes at multi-omic levels across 33 cancer types. Compared with other databases, such as Reactome, and The Human Protein Atlas, RHPCG focuses on the regulatory relationship among TFs, lncRNAs, circRNAs and Hippo-signaling-pathway genes by integrating 12 databases (Supplementary Table 1). Moreover, based on the high-throughput multi-omic datasets derived from TCGA, we managed to systematically analyze the molecular alterations of Hippo pathway genes in 33 cancer types using bioinformatic methods convenient for cancer researchers.

In conclusion, the RHPCG database can serve as a valuable resource for understanding the roles of Hippo signaling in carcinogenesis. The features of RHPCG can offer: (i) an overall view of the Hippo core genes in multiple omics; (ii) a quick search of Hippo regulatory motifs by mRNA, lncRNA, circRNA or miRNA; and (iii) visualization of all the results of Hippo-related motifs as a network that can be downloaded freely. In the future, we plan to add more datasets and investigate the cross talk with other tumor-related signaling pathways. We believe that RHPCG can help researchers gain an improved perspective of the alterations in the Hippo signaling pathway, as well as assist them in more efficiently filtering for the respective regulatory motifs. Moreover, we believe that RHPCG could also facilitate future studies in further exploring the biological molecular mechanisms in cancer. Thus, the RHPCG database could offer a new way in explaining how these motifs regulate the Hippo signaling pathway in cancer.

## Supplementary Material

Supplementary_table20191018_baz135

## Notes

**Database URL**: http://www.medsysbio.org/RHPCG
