# Supplementary material for: RHPCG: a database of the Regulation of the Hippo Pathway in Cancer Genome
Source: Database (Oxford). 2019 Dec 17;2019:baz135. doi: 10.1093/database/baz135 (PMC6917511; doi:10.1093/database/baz135)
Supplement: Supplementary_table20191018_baz135 [file supplementary_table20191018_baz135.docx]

**Supplementary Table 1.** The databases of regulatory relationship used in RHPCG.

| **Regulatory type** | **Database** | **Website** |
| --- | --- | --- |
| miRNA-gene | TargetScan | http://www.targetscan.org/ |
|  | miRTarBase | http://mirtarbase.mbc.nctu.edu.tw/ |
|  | miRanda | http://www.microrna.org/ |
|  | miRBase | http://www.mirbase.org/ |
| miRNA-circRNA | circbank | http://www.circbase.org/ |
|  | starBase v2.0 | http://starbase.sysu.edu.cn/ |
| miRNA-lncRNA | starBase v2.0 | http://starbase.sysu.edu.cn/ |
|  | miRcode | http://www.mircode.org/ |
| TF-lncRNA | ChIPBase v2.0 | http://deepbase.sysu.edu.cn/chipbase/ |
| TF-gene | MSigDB | http://software.broadinstitute.org/gsea/msigdb |
|  | TRANSFAC | http://gene-regulation.com/pub/databases.html |
| TF-miRNA | TransmiR | http://cmbi.bjmu.edu.cn/transmir |
|  | ChIPBase v2.0 | http://deepbase.sysu.edu.cn/chipbase/ |

**Supplementary Table 2.** Information of the 21 core genes in Hippo signaling pathway.

| **Gene Symbol** | **Reference** |
| --- | --- |
| AMOT | Liu, Y., Z. Lu, Y. Shi, et al. 2018 (PMID: 29217192); Li, Y., H. Zhou, F. Li, et al. 2015 (PMID: 26045165) |
| AMOTL1 | Liu, Y., Z. Lu, Y. Shi, et al. 2018 (PMID: 29217192); Li, Y., H. Zhou, F. Li, et al. 2015 (PMID: 26045165) |
| AMOTL2 | Liu, Y., Z. Lu, Y. Shi, et al. 2018 (PMID: 29217192); Li, Y., H. Zhou, F. Li, et al. 2015 (PMID: 26045165) |
| FRMD6 | Guan, C., Z. Chang, X. Gu, et al. 2019 (PMID: 31128910); |
| LATS1 | Pan, W.W., T. Moroishi, J.H. Koo, et al. 2019 (PMID: 30531839); Furth, N. and Y. Aylon 2017 (PMID: 28644436) |
| LATS2 | Pan, W.W., T. Moroishi, J.H. Koo, et al. 2019 (PMID: 30531839); Furth, N. and Y. Aylon 2017 (PMID: 28644436) |
| MOB1A | Sanchez-Vega, F., M. Mina, J. Armenia, et al. 2018 (PMID: 29625050); Couzens, A.L., S. Xiong, J.D.R. Knight, et al. 2017 (PMID: 28373298) |
| MOB1B | Sanchez-Vega, F., M. Mina, J. Armenia, et al. 2018 (PMID: 29625050); Couzens, A.L., S. Xiong, J.D.R. Knight, et al. 2017 (PMID: 28373298) |
| NF2 | Sanchez-Vega, F., M. Mina, J. Armenia, et al. 2018 (PMID: 29625050); Li, Y., H. Zhou, F. Li, et al. 2015 (PMID: 26045165) |
| RASSF1 | Vlahov, N., S. Scrace, M.S. Soto, et al. 2015 (PMID: 26549256); Kim, S.M., S. Ye, S.Y. Rah, et al. 2016 (PMID: 27230238); Levallet, G., C. Creveuil, L. Bekaert, et al. 2019 (PMID: 31055025) |
| RASSF6 | Levallet, G., C. Creveuil, L. Bekaert, et al. 2019 (PMID: 31055025); He, Z., T.T. Zhao, F. Jin, et al. 2018 (PMID: 29964010) |
| SAV1 | Sanchez-Vega, F., M. Mina, J. Armenia, et al. 2018 (PMID: 29625050); Hu, G., B. Dong, J. Zhang, et al. 2017 (PMID: 28938586); Bae, S.J., L. Ni, A. Osinski, et al. 2017 (PMID: 29063833) |
| STK3 | Sanchez-Vega, F., M. Mina, J. Armenia, et al. 2018 (PMID: 29625050); Zhou, D., C. Conrad, F. Xia, et al. 2009 (PMID: 19878874); Oka, T., V. Mazack and M. Sudol 2008 (PMID: 18640976) |
| STK4 | Sanchez-Vega, F., M. Mina, J. Armenia, et al. 2018 (PMID: 29625050); Zhou, D., C. Conrad, F. Xia, et al. 2009 (PMID: 19878874); Oka, T., V. Mazack and M. Sudol 2008 (PMID: 18640976) |
| TEAD1 | Chai, J., S. Xu and F. Guo 2017 (PMID: 28483529) |
| TEAD2 | Sanchez-Vega, F., M. Mina, J. Armenia, et al. 2018 (PMID: 29625050); |
| TEAD3 | Noland, C.L., S. Gierke, P.D. Schnier, et al. 2016 (PMID: 26724994) |
| TEAD4 | Kang, W., T. Huang, Y. Zhou, et al. 2018 (PMID: 29367737) |
| WWC1 | Sanchez-Vega, F., M. Mina, J. Armenia, et al. 2018 (PMID: 29625050) |
| TAZ | Sanchez-Vega, F., M. Mina, J. Armenia, et al. 2018 (PMID: 29625050); Kodaka, M. and Y. Hata 2015 (PMID: 25266986); Hansen, C.G., T. Moroishi and K.L. Guan 2015 (PMID: 26045258) |
| YAP1 | Sanchez-Vega, F., M. Mina, J. Armenia, et al. 2018 (PMID: 29625050); Kodaka, M. and Y. Hata 2015 (PMID: 25266986); Hansen, C.G., T. Moroishi and K.L. Guan 2015 (PMID: 26045258) |
